# Proteomic study of hypothalamus in pigs exposed to heat stress

**DOI:** 10.1186/s12917-020-02505-1

**Published:** 2020-08-12

**Authors:** Tian-yue Yu, Yan-hong Yong, Jun-yu Li, Biao Fang, Can-ying Hu, Lian-yun Wu, Xiaoxi Liu, Zhichao Yu, Xingbin Ma, Yadnyavalkya Patil, Ravi Gooneratne, Xiang-hong Ju

**Affiliations:** 1 Department of Veterinary Medicine, Guangdong Ocean University, Zhanjiang, 524088 China; 2 Shenzhen Institute of Guangdong Ocean University, Shenzhen, 518018 China; 3 Department of Animal Science, Guangdong Ocean University, Zhanjiang, 524088 China; 4 Faculty of Agriculture and Life Sciences, Lincoln University, Lincoln, 7647 New Zealand

**Keywords:** Pigs, Heat stress, Hypothalamus, Quantitative proteomics

## Abstract

**Background:**

With evidence of warming climates, it is important to understand the effects of heat stress in farm animals in order to minimize production losses. Studying the changes in the brain proteome induced by heat stress may aid in understanding how heat stress affects brain function. The hypothalamus is a critical region in the brain that controls the pituitary gland, which is responsible for the secretion of several important hormones. In this study, we examined the hypothalamic protein profile of 10 pigs (15 ± 1 kg body weight), with five subjected to heat stress (35 ± 1 °C; relative humidity = 90%) and five acting as controls (28 ± 3 °C; RH = 90%).

**Result:**

The isobaric tags for relative and absolute quantification (iTRAQ) analysis of the hypothalamus identified 1710 peptides corresponding to 360 proteins, including 295 differentially expressed proteins (DEPs), 148 of which were up-regulated and 147 down-regulated, in heat-stressed animals. The Ingenuity Pathway Analysis (IPA) software predicted 30 canonical pathways, four functional groups, and four regulatory networks of interest. The DEPs were mainly concentrated in the cytoskeleton of the pig hypothalamus during heat stress.

**Conclusions:**

In this study, heat stress significantly increased the body temperature and reduced daily gain of body weight in pigs. Furthermore, we identified 295 differentially expressed proteins, 147 of which were down-regulated and 148 up-regulated in hypothalamus of heat stressed pigs. The IPA showed that the DEPs identified in the study are involved in cell death and survival, cellular assembly and organization, and cellular function and maintenance, in relation to neurological disease, metabolic disease, immunological disease, inflammatory disease, and inflammatory response. We hypothesize that a malfunction of the hypothalamus may destroy the host physical and immune function, resulting in decreased growth performance and immunosuppression in heat stressed pigs.

## Background

Pigs are homeotherms with under-developed sweat glands. Therefore, at high temperature, pigs experience stress more than other species [1]. Heat stress (HS) affects pigs markedly, including changes to the metabolism of several organ systems [2], deposition of fat [3], disruption to energy balance [4], and lowering of meat quality [5]. Bell reported that during HS, the barrier function of the intestinal mucosa may be compromised due to increased hypoxia and formation of free radicals in pig visceral organs [6]. Ju et al. reported that HS alters immune and biochemical indexes leading to metabolic and endocrine disorders in Bama miniature pigs [7]. Now, more than ever, it is important to understand the susceptibility of livestock to stress induced diseases in relation to immune system functioning and understand the mechanism behind such diseases, because most emerging animal diseases are seriously threatening public health.

The hypothalamic–pituitary–adrenal axis is the most important pathway in the physiological control of stress [8]. Its activation starts with the secretion of corticotropin-releasing hormone from the hypothalamic paraventricular nucleus; this promotes the release of adrenocorticotropic hormone from the pituitary, which in turn releases glucocorticoids from the adrenal cortex [9]. Sutherland et al. observed a decrease in levels of plasma cortisol (a major glucocorticoid) in pigs exposed to HS for 21 days [10]. Kataria detected significantly higher levels of serum prolactin and cortisol in pigs exposed to a higher temperature (45°–46 °C) [11]. In cattle, cortisol increased in the early stages of HS but subsequently returned to normal levels [12]. Hence, the objective of our study was to examine the function and interaction of deferentially expressed proteins (DEPs) in the hypothalamus of pigs subjected to HS. This would provide the basis for further study of the mechanism of HS effects on the physiological function and metabolism of the hypothalamus.

Isobaric tags for relative and absolute quantification (iTRAQ) technology is a comparative proteomic science developed in recent years [13]. It has been used to describe proteomic analysis of the hypothalamic response during sleep regulation [14], feeding patterns [15], microgravity conditions [16], anesthesia [17], exposure to environmental toxicants [18], and reproductive cycles [19]. In previous studies, when the ambient temperature was maintained at 35 ± 1 °C and the relative humidity was maintained at 90%, the body temperature of pigs was shown to increase by 0.5 to 1°, and the respiratory rate also increased significantly [20]. In this study, we used iTRAQ technology to study proteomic changes in the hypothalamus of pigs exposed to HS for 7 days to determine the mechanism of immune regulation and to provide a theoretical basis for the control and prevention of HS.

## Results

### Clinical changes caused by heat stress

**Fig. 1:**
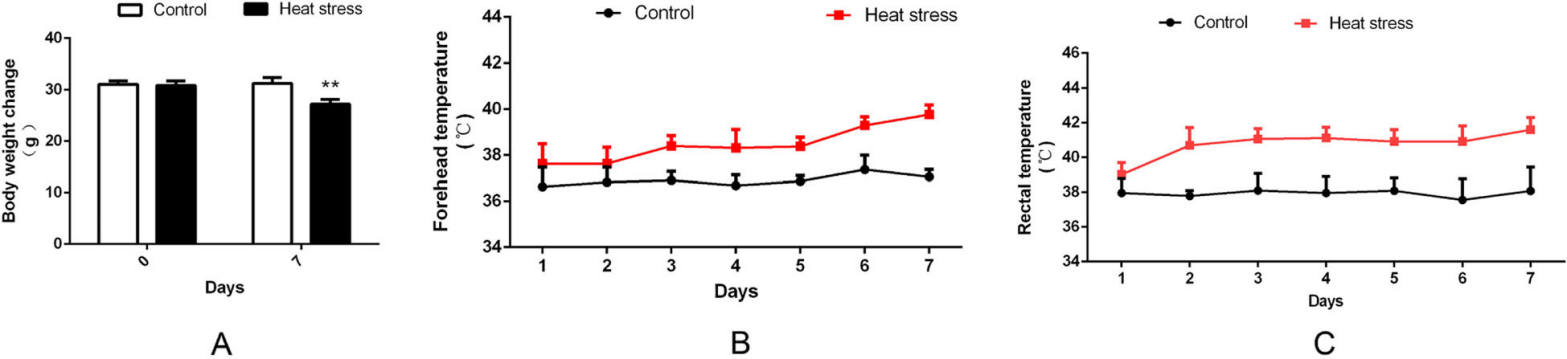
Clinical changes caused by heat stress. (**a**) Changes in pig body weight, (**b**) forehead temperature, and (**c**) rectal temperature, under heat stress

We measured the body weight, rectal temperature, and forehead temperature of pigs on days 1 to 7 of implementing the heat stress treatment. The body weight of pigs was significantly reduced on day 7 of heat stress (Fig. 1a). Under heat stress conditions, both forehead temperature and rectal temperature of pigs were higher than control, the most difference between heat stressed and control pigs appearing on day 7 (Fig. 1b, c). These results indicated that heat stress significantly affected the growth performance (in terms of the above-mentioned traits) of pigs.

### iTRAQ-based DEP identification and quantitative analysis of swine hypothalamic tissue

**Fig. 2:**
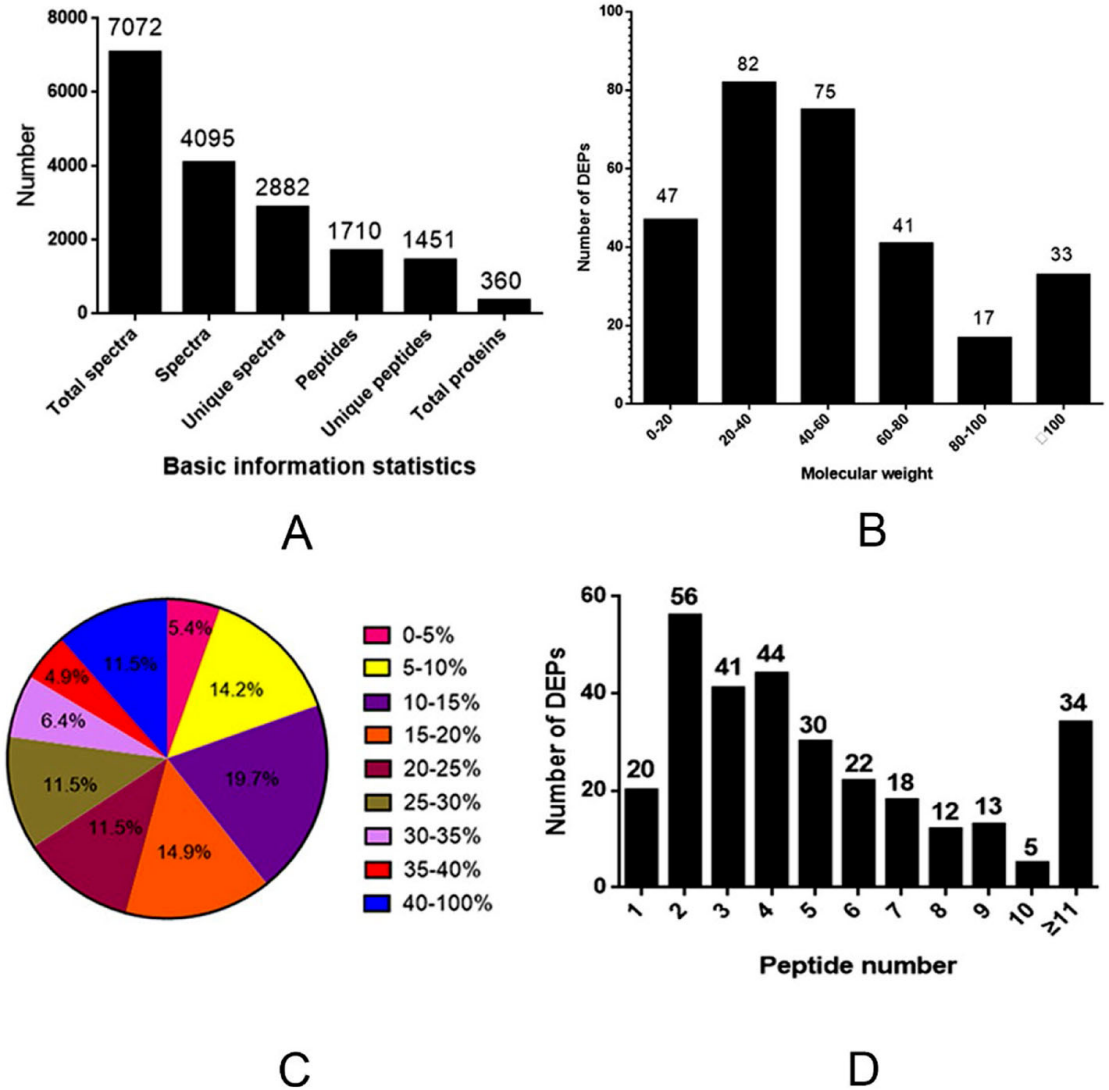
Protein identification in the hypothalamus of heat-stressed (HS) and control pigs. (**a**) Numbers of proteins identified, (**b**) distribution of the DEPs among molecular weight (kD) classes, (**c**) coverage of DEPs by the identified peptides, and (**d**) distribution of DEPs containing different numbers of identified peptides

All protein and peptide identifications were obtained by database searching and stringent data filtering. The LC–MS/MS analysis produced 7072 spectra, corresponding to 2882 unique peptides; 360 proteins were identified at a false discovery rate (FDR) of ≤0.01 (Fig. 2a). According to the level of protein abundance, proteins were regarded as different proteins when the difference multiple reached 1.5 or more, and the statistical test showed significant difference (*p* < 0.01). Thus, there were 295 DEPs identified in the hypothalamus, of which 148 were up-regulated and 147 were down-regulated. As shown in Table 1, 40 key DEPs were selected, including information on 20 up-regulated proteins and 20 down-regulated proteins. The information of all the determined DEPs is shown in Supplementary Table 1.

**Table 1:** The differentially expressed proteins from hypothalamus in pigs under HS

Protein name	Accession number	Peptides	Ratio (Heat stress/Control)	Functions
***Down-regulated in Hypothalamus***				
Heat shock 70 kDa protein 12A	gi|350,593,095	7	0.439	ATP binding
Similar to V-type proton ATPase subunit E 1 isoform 2	gi|350,584,473	6	0.513	hydrogen-exporting ATPase activity, phosphorylative mechanism
HPCA	gi|115,394,790	7	0.151	actin binding and calcium ion binding
Heat shock 90kD protein 1, beta	gi|346,986,428	15	0.666	GTP, CTP binding and UTP binding, chaperones
Similar to protein kinase C and casein kinase substrate in neurons protein 1-like	gi|335,292,069	7	0.179	cytoskeletal protein binding
Tubulin polymerization promoting protein p25 alpha	gi|170,178,280	7	0.225	microtubule binding and calcium ion binding
Similar to annexin A6-like	gi|350,594,505	10	0.611	calcium ion binding
Similar to tubulin alpha-4A chain	gi|335,303,414	18	0.161	protein binding and GTP binding
Na+/K+ transporting alpha 3 polypeptide	gi|283,443,672	25	0.282	metal ion binding and sodium: potassium-exchanging ATPase activity
Similar to pyruvate kinase isozymes M1/M2 isoform 1	gi|194,038,728	17	0.4	pyruvate kinase activity
Similar to guanine nucleotide-binding protein G(I)/G(S)/G(T) subunit beta-2-like isoform 2	gi|311,251,041	5	0.474	signal transducer activity
Similar to erythrocyte membrane protein band 4.1-like 1 isoform 1	gi|335,304,751	9	0.601	actin binding and structural molecule activity
Tubulin beta-2B chain	gi|343,478,189	16	0.154	structural molecule activity
Clathrin heavy chain	gi|224,492,556	38	0.362	ankyrin binding and structural molecule activity
Similar to contactin-1-like isoform 2	gi|350,584,500	12	0.343	glycoprotein binding and carbohydrate binding
hexokinase 1	gi|342,187,282	15	0.637	hexokinase activity
Similar to synaptic vesicle glycoprotein 2A	gi|194,036,298	6	0.325	Receptor and transmembrane transporter activity
Galectin-1	gi|47,716,872	7	0.431	signal transducer activity and laminin binding
Synapsin Ib	gi|212,525,788	13	0.205	ATP binding and actin binding
Similar to tubulin alpha-1D chain	gi|194,043,861	17	0.121	GTP binding and protein heterodimerization activity
***Up-regulated protein in hypothalamus***				
Annexin A2	gi|52,631,987	14	2.322	phosphatidylinositol-4,5-bisphosphate binding
Hsp27	gi|55,668,280	6	2.771	protein kinase C binding and protein kinase C inhibitor activity
Similar to annexin A11	gi|194,042,189	6	2.757	serine-type endopeptidase inhibitor activity
Similar to prohibitin	gi|3a50590415	6	1.519	sequence-specific DNA binding RNA polymerase II transcription factor activity
Similar to glyoxalase domain-containing protein 4-like	gi|335,298,275	7	1.616	Amino acid transport and metabolism
Stress-70 protein, mitochondrial	gi|311,250,237	16	2.105	ATP binding and unfolded protein binding, testosterone 17-beta-dehydrogenase (NADP+)
Similar to protein disulfide-isomerase A4-like	gi|311,264,773	11	1.889	electron carrier activity and protein binding
long-chain 3-ketoacyl-CoA thiolase	gi|6,165,556	7	3.162	NAD binding and long-chain- enoyl-CoA hydratase activity
Similar to ezrin	gi|350,578,005	9	4.869	cell adhesion molecule binding and actin filament binding
Gastrin-binding protein	gi|433,066	9	2.614	acetyl-CoA C-acetyltransferase activity
Ppk 98; a protein kinase	gi|431,944	20	1.783	virion and calcium ion binding
78 kDa glucose-regulated protein	gi|350,579,657	22	1.634	chaperone binding and unfolded protein binding
Heat shock 10kD protein	gi|30,525,868	7	10.249	chaperone binding
Similar to caldesmon	gi|311,275,365	5	2.353	Calmodulin, actin and myosin binding
Similar to annexin A5	gi|335,293,906	12	2.117	calcium ion binding; binding, bridging
Similar to annexin A11	gi|194,042,189	6	2.757	serine-type endopeptidase inhibitor activity
Similar to selenium-binding protein 1	gi|194,036,227	20	2.677	selenium and protein binding
Non-selenium glutathione phospholipid hydroperoxide peroxidase (PHGPx)	gi|6,689,393	7	2.148	glutathione peroxidase activity
Similar to serpin A3–8	gi|350,587,171	10	4.428	serine-type endopeptidase inhibitor activity
Glucosidase 2 subunit beta precursor	gi|347,446,687	6	1.733	phosphatidylinositol binding

The molecular weights of most DEPs were in the range of 20–60 kD (157 DEPs) (Fig. 2b). In addition, the identified DEPs had high peptide coverage, of which 80 and 54% showed more than 10 and 20% sequence coverage, respectively (Fig. 2c). About 74% of the identified DEPs had three or more peptides (Fig. 2d).

### Subcellular localization and canonical pathways of identified DEPs

**Fig. 3:**
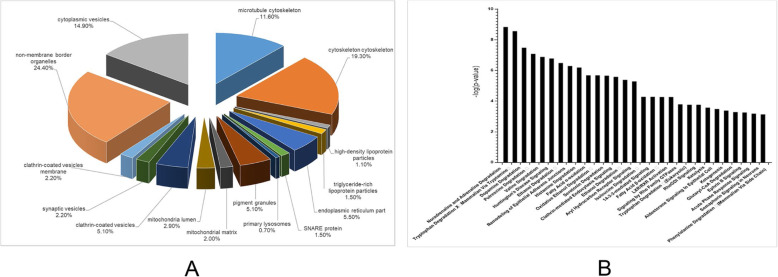
Bioinformatics analysis of the DEPs identified in the hypothalamus of pigs. **a** Subcellular locations of the proteins with differential expression in heat-stressed (HS) and control pigs. **b** Thirty most related canonical pathways from Ingenuity Pathway Analysis

To elucidate the functional characteristics of DEPs in the hypothalamus under HS, the DEPs of the hypothalamic tissues were analyzed based on the basic biological functions, clustering of molecular functions, and cell locations of proteins in the UniProtKB/Swiss-Prot, TrEMBL, and Gene Ontology databases. The DEPs identified from hypothalamic tissues are altered by heat stress and are located in different subcellular areas, mainly in cytoplasmic vesicles (14.9%), cytoskeleton (19.3%) and non-membrane border organelles (24.4%) (Fig. 3a).

To better understand these DEPs, further analysis was undertaken using the Ingenuity Pathway Analysis (IPA) tool. Canonical pathways were examined first; the top 30 pathways are shown in Fig. 3b, including pathways related to inflammation and immunity, such as Huntington’s disease signaling, clathrin-mediated endocytosis signaling, and Liver X receptor/Retinoic X receptor activation (Fig. 3b).

### Functional characteristics and bioinformatics analysis of DEPs

**Fig. 4:**
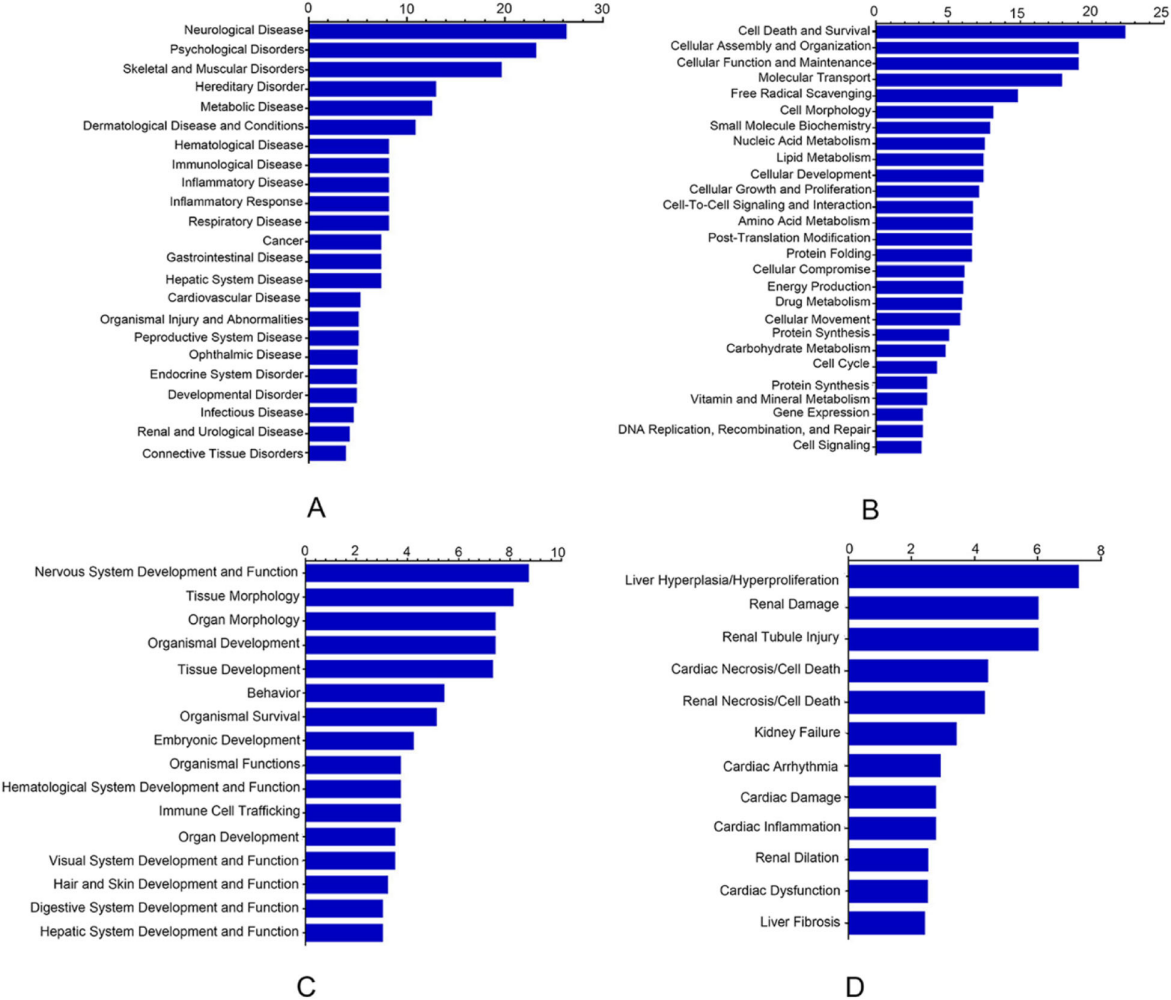
Functional characterization of DEPs in the hypothalamus of pigs under heat stress. (**a**) diseases and disorders, (**b**) molecular and cellular functions, (**c**) physiological system development and functions, and (**d**) toxicological functions

Compared with the human genome database, annotation in the pig genome database is relatively scarce, and many protein features are not identified or classified. The differential proteins identified in our study were converted to the human protein gene bank identification (gi) number. The gi numbers and regulatory levels of these proteins were entered into the IPA software, and based on the database, protein–protein interaction signal pathways were constructed. The proteins identified by iTRAQ in the hypothalamus were clustered according to different functions; four statistically significant functional groups were found, namely, diseases and disorders, molecular and cellular functions, physiological and phylogenetic functions, and toxicological functions (Fig. 4).

The DEPs identified in the hypothalamus of pigs under HS were consistent and corresponded to 23 diseases and disorders including proteins that are related to neurological diseases, psychological disorders, skeletal and muscular disorders, hereditary disorders, metabolic diseases, dermatological diseases and conditions, hematological diseases, immunological diseases, and inflammatory diseases, among others (Fig. 4a). The DEPs could also be assigned to 27 molecular and cellular functions groups, including cell death and survival, cell assembly and organization, cell function and maintenance, molecular transport, free radical scavenging, cell morphology, small molecule biochemistry, nucleic acid metabolism, lipid metabolism, and cellular development (Fig. 4b); 16 physiological system development and functions groups, including nervous system development and function, tissue morphology, organ morphology, organismal development, tissue development, behavior, organismal survival, embryonic development, organismal function, hematological system development and function, immune cell trafficking, and organ development (Fig. 4c); and 12 toxicity functions groups, namely, liver hyperplasia/hyper-proliferation, renal damage, renal tubule injury, cardiac necrosis/cell death, kidney failure, cardiac arrhythmia, cardiac damage, cardiac inflammation, renal dilation, cardiac dysfunction, liver fibrosis (Fig. 4d).

**Fig. 5:**
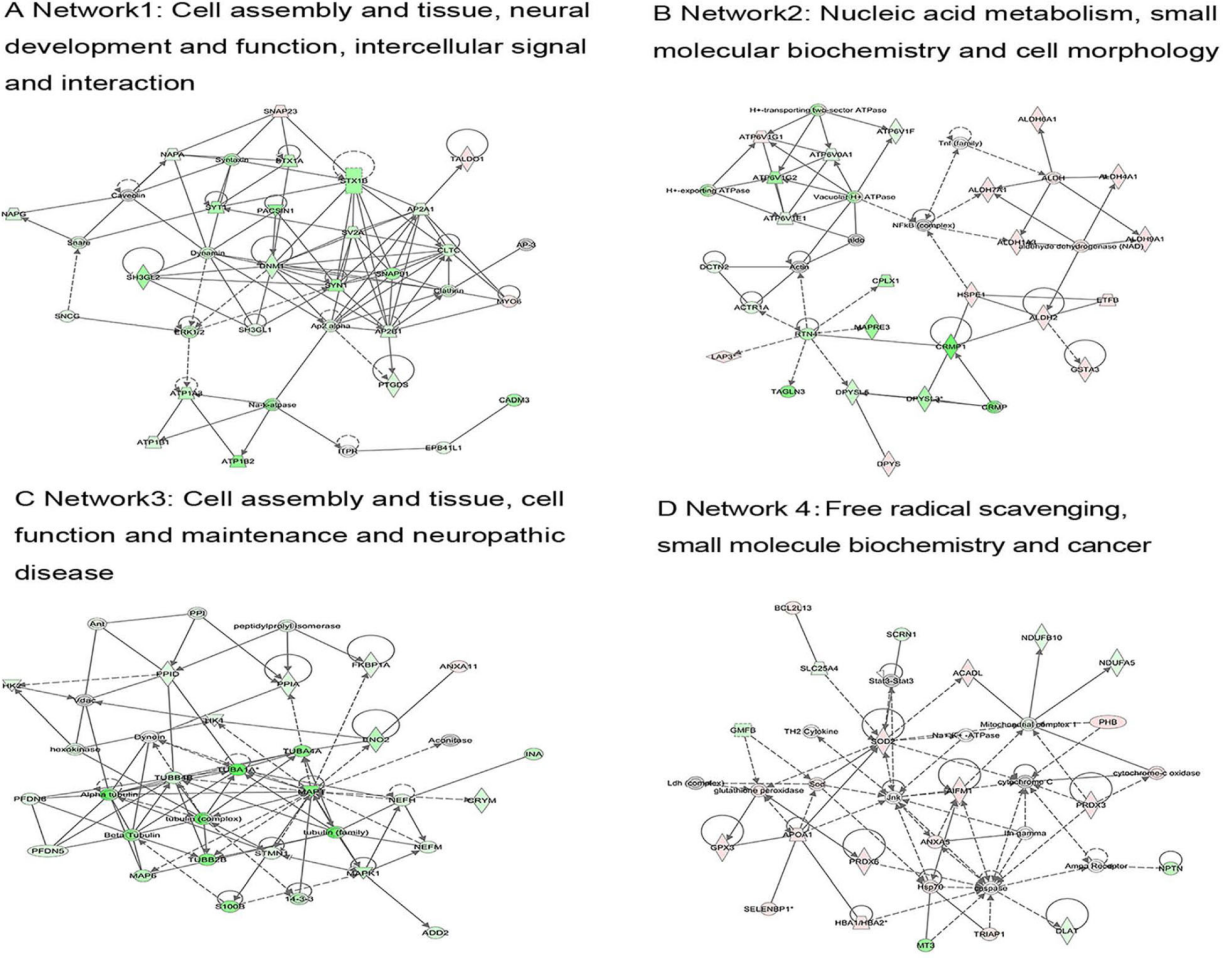
Ingenuity Pathway Analysis of differentially expressed proteins in the hypothalamus of pigs under heat stress. Red is up-regulated, green is down-regulated, and white is a protein that is involved in pathway regulation but not identified in this study. A darker color indicates a greater change in the expression level of the protein. Different shapes represent different molecular types (e.g., protein families). The lines connecting the molecules represent intermolecular relationships—the dashed lines are indirect effects and the solid lines are direct effects. The arrows represent specific molecular relationships and directions of action

Among the DEPs identified in the hypothalamus, 13 functional networks were constructed (Fig. 5). The four networks of interest correspond to (1) Cell assembly and tissue, neural development and function, intercellular signal and interaction (Fig. 5a); (2) Nucleic acid metabolism, small molecular biochemistry, and cell morphology (Fig. 5b); (3) Cell assembly and tissue, cell function and maintenance, and neuropathic disease (Fig. 5c); (4) Free radical scavenging, small molecule biochemistry, and cancer (Fig. 5d). Proteins that are present in these pathways and identified as up-regulated DEPs in our analysis are depicted in shades of red and those identified as down-regulated are shown in green. Proteins in the network, but not identified as DEPs in our study, are depicted in white.

### Validation of protein identification and quantification

**Fig. 6:**
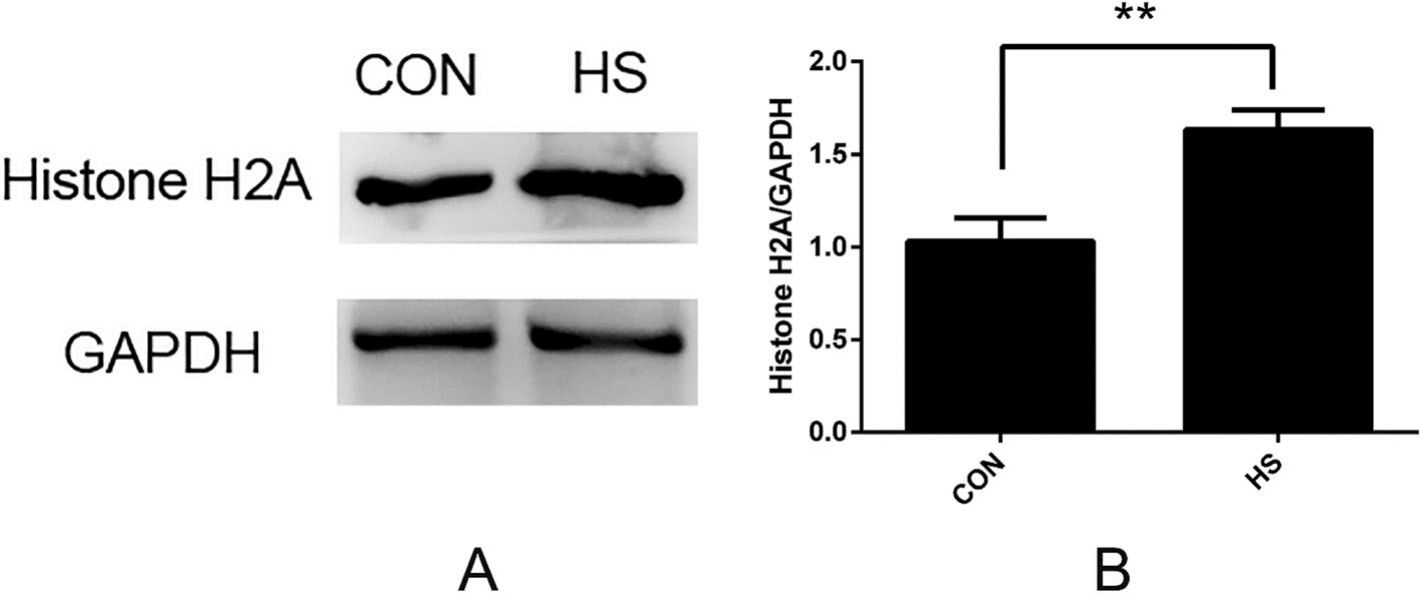
Western blotting identification of DEPs. **a** Expression of Histone H2A in the hypothalamus of pigs at day 7 of heat stress. Con = control pigs; HS = heat-stressed pigs. **b** Expression of Histone H2A was significantly increased in the HS group compared with the control group

Histone H2A is a type of innate immune molecule discovered in recent years that plays a key role in the phagocytosis of neutrophils and in the clearance of pathogenic microorganisms. To verify the reliability of DEPs identified by iTRAQ, the hypothalamus of pigs on day 7 of HS was used to detect the expression of Histone H2A by western blotting. As shown in Fig. 6 (The complete picture was shown in additional Fig. 1), compared with the control group, the expression level of Histone H2A in the HS group was significantly up-regulated (*p* < 0.01), which was in agreement with the results of the iTRAQ analysis.

## Discussion

Under stress conditions, endocrine factors such as glucocorticoids and catecholamines can cause immune cell apoptosis, lymphocyte proliferation damage and dendritic cell dysfunction [21, 22], thereby inducing immunosuppression in animals. The body’s stress system consists of the hypothalamic–pituitary–adrenal axis, the sympathetic nervous system, and adrenal medulla. The hypothalamus is a vital subcortical center and plays a crucial role in maintaining homeostasis in the body, such as the body’s temperature regulation, the balance of water and salt, blood pressure stability, reproductive function integration and regulation of a variety of stress responses. It is important to elucidate the changes in the proteomic of the hypothalamus of heat stressed pigs, as this will facilitate the understanding of the mechanism of immunosuppression in the host. In this study, we identified 295 DEPs in the hypothalamus of heat stressed pigs using iTRAQ technology. The DEPs were mainly distributed in cytoplasmic vesicles, cytoskeleton and non-membrane border organelles, and caused cell death, and immunological and inflammatory diseases. This laid the foundation for systematically revealing the regulatory mechanism of stress-induced immunosuppression.

NADH (Nicotinamide adenine dinucleotide) reacts with free radicals to inhibit lipid peroxidation and protect mitochondrial membranes and mitochondrial function. Studies have found that NADH can reduce the oxidative stress of cells caused by various factors such as radiation, drugs, toxic substances, strenuous exercise, ischemia, etc., thereby protecting vascular endothelial cells, liver cells, cardiomyocytes, fibroblasts, and neurons [23]. Research by He et al. showed that iTRAQ technology identified 212 different proteins in duck meat under heat stress conditions, of which 84% of down-regulated proteins involved enzymes that regulated the redox reaction process, such as COXs, NADH metabolism, and ATP synthesis/binding related enzymes [24]. The 147 down-regulated proteins in the pig hypothalamus identified in this study include the NADH protein, so it can be concluded that the heat-stressed mitochondrial function and energy supply in the pig hypothalamus caused oxidative damage to the cells.

The heat shock protein (HSP) response is a highly conserved cellular response to external stress in all species. HSPs, including heat shock protein 27 (HSP27), take part in antigen presentation, intracellular trafficking, apoptosis, and act as molecular chaperones by assisting nascent polypeptides in assuming their proper conformations [25]. HSP27 is a multidimensional protein that acts as a protein chaperone and an antioxidant, and plays a role in the inhibition of apoptosis and actin cytoskeletal remodeling [26]. In this study, HSP27 was up-regulated in pig hypothalamus under HS (Table 1), further indicating the role of HSP27 in hypothalamic injury and highlighting its relevance to inflammatory responses.

Histone H2A plays a role in many cell activities such as gene silencing, cell cycle regulation, fatty acid metabolism and apoptosis [27]. Sandra found that Histone H2A was significantly up-regulated in Bacille Calmette-Guerin (BCG)-inoculated mice, indicating that Histone H2A plays a crucial role in inflammation [28]. Nakata found that heat shock could significantly increase the expression of Histone H2A in Hela cells [29]. In this study, up-regulated Histone H2A was found in DEPs of heat-stressed pigs. However, Duff’s research on bovine respiratory disease found that Histone H2A was down-regulated in immunosuppressed bovine neutrophils [12]. Hence, in HS, whether the change of H2A is related to immunosuppression requires further study. DEPs, along with 13 proteins including Annexin A2 and 17β-HSD, proteins were down-regulated in the hypothalamus of heatstroke rats [30], and a majority of these proteins was also found in the hypothalamus of HS pigs. These proteins play a key role in cell migration, free radical scavenging, maintenance of cytoskeleton, organ morphology, and apoptosis.

Das found that heat stress induces Huntington’s disease in mice, and HYPK (Huntingtin Yeast Partner K) could alleviate huntington’s reaction by suppressing the heat shock response [31]. Clathrin-mediated endocytosis is a type of receptor-mediated phagocytosis that delivers foreign substances from the cell membrane to the endosome, and is used by many viruses—for example, influenza virus [32], hepatitis C virus [33], and Bovine viral diarrhea virus [34]—to complete the cell invasion process. Fujita et al. found that HSP27 phosphorylation could maintain actin stability, thereby stabilizing the cytoskeleton and inhibiting apoptosis to some extent [35]. In this study, functional characterization of DEPs showed that Huntington’s disease signaling and clathrin-mediated endocytosis signaling may play a role in HS induced physiological disorder in the host. The proportion of DEPs affected by heat stress in cytoskeleton was 19.3%; HSP27 also showed a downward trend in the hypothalamus, suggesting that heat stress affected the function and maintenance of cells, and induced neurological diseases.

Overall, the IPA showed that these DEPs take part in cell death and survival, cellular assembly and organization, cellular function and maintenance, and are significantly correlated to neurological diseases, metabolic diseases, immunological diseases, inflammatory diseases, and inflammatory responses. These changes may affect the normal functioning of the hypothalamus and result in the faulty regulation of physiology and immune function of the host. Consequently, a decrease in clinical performance, including decrease in body weight, feed consumption, and immunosuppression, may appear in heat stressed pigs.

## Conclusion

In this study, heat stress significantly increased the body temperature and reduced daily gain in pigs. Furthermore, we identified 295 differentially expressed proteins, 147 of which were down-regulated and 148 up-regulated in the hypothalamus of heat stressed pigs. The IPA showed that these DEPs took part in cell death and survival, cellular assembly and organization, cellular function and maintenance, and were related to neurological diseases, metabolic diseases, immunological diseases, inflammatory diseases, and inflammatory responses. The negative effect on hypothalamus function may impair the physical and immune function of the host, resulting in the decrease of growth performance and immunosuppression in heat stressed pigs.

## Methods

Pig feeding and experimentation were carried out in accordance with the National Institutes of Health (NIH) guidelines for the care and use of laboratory animals. Pigs were raised and studied according to the guidelines of the National Institutes of Health (NIH) on raising and using experimental animals. All projects have been approved by the Animal Care and Use Committee of Guangdong Ocean University, China.

### Experimental animals and groups

The castrated Bama miniature pigs were purchased from the Bama mini-pig farm in Bama Miniature Pigs Breeding-farm in Guangxi Zhuang Autonomous Region, China, about 3 months, and weighed 15 ± 1 kg. They were randomly divided into control group (*n* = 5) and heat stress group (n = 5) according to one-way ANOVA. The air temperature of the control pigs was 28 ± 3 °C, while the temperature of the HS group was maintained at 35 ± 1 °C. The pigs in the control group and the heat stress group were kept separately. The relative humidity for both groups was 80–90%. All pigs had free access to water. The basic diet (see the digestible energy Table 2) was based on the recommended nutritional composition of the pig breed. Pigs were fed twice a day, once in the morning and once in the evening.

**Table 2:** The digestible energy of Bama miniature pigs

Feed ingredients	Ingredients /%	Nutrition level	Ingredients
Corn	70	Digestive energy^②^/(MJ•kg^−1^)	14.23
Soybean meal	10	CP/%	14.00
Extruded soybean	5	Ca/%	0.50
Rapeseed meal	5	P/%	0.43
Soybean oil	4.8	Lysine/%	0.88
Limestone	1.2		
Premix^①^	4		

①The premix is provided for each kilogram of full price material: Vitamin A 18000 IU, Vitamin D 34000 IU, Vitamin E 100 mg, Vitamin B2 10 mg, Vitamin B1 4 mg, Vitamin B12 40 mg, biotin 200 mg, pantothenic acid 20 mg, niacin 15 mg, iron 100 mg, copper 30 mg, zinc 110 mg, manganese 20 mg, selenium 0.67 mg, cobalt 1 mg;

②Digestive energy is calculated value, others are measured value

### Hypothalamus collection

Pigs were euthanized by a head-only electric stun tong apparatus on the 7th day under HS, followed by manual exsanguination. Immediately after slaughter, the hypothalamus tissue was removed. Subsequently, the tissue was washed with PBS (phosphate buffer saline) to remove any blood and contaminants on the surface of the tissue. The hypothalamic tissue was placed into sterile tubes and snap frozen in liquid nitrogen. In the laboratory, the frozen specimens were stored at − 80 °C until biochemical and molecular analysis.

### Protein extraction and quantification, iTRAQ labeling, and strong Cation exchange (SCX) fractionation

Frozen hypothalamus tissue from control and heat stress were exposed to liquid nitrogen and were ground into fine powder using a mortar. The powder (~ 100 mg per pig) was transferred to sterile tubes with 1 mL of lysis buffer (pH 8.5; containing 7 m urea, 2 m thiourea, 4% CHAPS, 40 mm Tris-HCl, 10 mm dithiothreitol) for 5 min. Then, the tissue was homogenized with an Ultrasonic Cell Disruptor (VCX130, USA) at 20% power output for 10 min with 2/4-s on/off cycles. Following this, the digest was centrifuged at 25,000×*g* for 30 min at 4 °C, and the supernatant was stored at − 80 °C for further analysis.The protein concentration in the supernatant was determined using the 2-D Quant Kit (Thermo Fisher Scientific, MA, USA). Protein (100 μg) from PAMs was precipitated with acetone overnight at − 20 °C and dissolved using the iTRAQ dissolution buffer. After reduction and alkylation as described in the iTRAQ protocol (Applied Biosystems), protein solutions were digested overnight at 37 °C with sequence grade modified trypsin (Promega) and then labeled with the iTRAQ tags as described in the iTRAQ protocol. Subsequently, labeled peptides were combined and fractionated by cation exchange (SCX) chromatography [38] and desalted on C18 Cartridges (66872-U; Sigma, St. Louis, MO, USA). The dried peptide mixture was reconstituted and acidified with 2 mL of buffer A (10 mM KH_2_PO_4_ in 25% of CAN; pH 3.0) and loaded onto a polysulphoethyl 4.6 × 100 mm column (5 μm, 200 Å, PolyLC Inc., Maryland, USA). The peptides were eluted at a flow rate of 1 mL/min with a gradient of 0–5% buffer B (2 m KCl, 10 mm KH2PO4 in 25% of acetonitrile; pH 2.7) for 5 min, 5–10% buffer B for 10–15 min, 10–30% buffer B for 25–35 min, and 30–50% buffer B for 35–50 min. The elution was monitored at A_214_, and fractions were collected each minute. The collected fractions (about 30 fractions) were finally combined into 10 pools and desalted on C18 Cartridges (Empore™ SPE Cartridges C18 (standard density), 7 mm bed I.D., 3 ml volume, Sigma). Each fraction was concentrated by vacuum centrifugation and reconstituted in 40 μL of 0.1% (v/v) trifluoroacetic acid. All samples were stored at − 80 °C until nanoLC-MS/MS analysis was conducted.

### LC-MS/MS analysis

Each fraction was injected for nanoLC-MS/MS analysis. The peptide mixture was loaded onto a reverse phase trap column (Thermo Scientific Acclaim PepMap100, 100 μm × 2 cm, nanoViper C18) connected to the C18-reverse phase analytical column (Thermo Scientific Easy Column, 10 cm long, 75 μm inner diameter, 3 μm resin) in buffer A (0.1% formic acid) and separated with a linear gradient of buffer B (84% acetonitrile and 0.1% formic acid) at a flow rate of 300 nL/min controlled by IntelliFlow technology. Peptides eluted by high performance liquid chromatography were directly injected into the Q-Exactive mass spectrometer (Thermo Fisher Scientific, MA, USA). Data were acquired in the positive ion mode with a selected mass range of 300–1800 mass/charge (m/z) [36]. Q-Exactive survey scans were obtained at 70,000 (m/z 200) and 17,500 (m/z 200), with the resolution for higher-energy collisional dissociation spectra and maximum ion injection times fixed at 20 and 60 MS, respectively. Dynamic exclusion (40.0 s duration) was used. MS/MS data were collected using the top 10 most abundant precursor ions. The normalized collision energy was 30 eV, and the underfill ratio was defined as 0.1%. The instrument was run with the peptide recognition mode enabled.

### iTRAQ data analysis

Protein identifications were performed using the MASCOT engine (version 2.3.02; Matrix Science, London, UK) embedded in Proteome Discoverer 1.4 (Thermo Fisher Scientific). The search parameters were as follows: (1) database, uniprot; (2) taxonomy, *Homo sapiens*; (3) Enzyme, trypsin; (4) fixed modifications, carbamidomethyl of C, iTRAQ 4plex (N-term), iTRAQ 4plex (K); (5) variable modifications, oxidation of M; (6) max missed cleavages, 2; (7) peptide charges state, + 2, + 3, and + 4; (8) peptide mass tolerance, 20 ppm; (9) mass/mass tolerance, ±0.05 Da. Differentially expressed proteins were defined as those that were different by at least 2-fold between two groups. The data analysis was supported by Wayen Biotechnologies. (Shanghai, China).

### Bioinformatics analysis

Go numbers of all significantly regulated proteins and some unaltered proteins were imported into the Ingenuity Pathway Analysis software (IPA, www.ingenuity.com) for bioinformatics analysis based on published reports and databases such as Gene Ontology, Uniprot, and TrEMBL. The canonical pathways and protein interaction networks of the DEPs were analyzed using the IPA.

### Western blotting

Pig hypothalamus samples from both HS and control groups were homogenized in Radio-Immunoprecipitation Analysis buffer (Thermo Fisher Scientific, MA, USA), followed by ultrasonication on ice. Homogenates were then incubated on ice for 20 min and centrifuged at 12,000×*g* for 15 min at 4 °C. Supernatants were collected and total protein concentration measured using the BCA assay. Next, protein degeneration was performed at 100 °C for 10 min. Proteins were separated on SDS-PAGE gels, then transferred to PVDF (polyvinylidene fluoride) membranes and blocked in 5% non-fat dry milk/TBST solution at 37 °C room temperature for 2 h. Membranes were incubated overnight at 4 °C in western blotting diluent containing primary antibodies: histone H2A (1:1000; Abcam, Cambridge, UK), and GAPDH (1:1000; Cell Signaling Technology, America). After washing them four times with TBST, membranes were incubated in blocking solution containing anti-rabbit secondary antibodies (1:1000) at 37 °C for 1 h. Blots were visualized using an ECL detection system and proteins were quantified using a ChemiDoc XRS+ image analyzer (Bio-Rad, Hercules, CA, USA). Densitometric analysis was used to evaluate the expression levels of target proteins. Ratios of densitometric measurements of target proteins relative to GAPDH were compared between control and HS groups. Three experimental replicates were performed, and independent Student’s t-test was used to assess statistical difference.

### Statistical analysis

Statistical analysis was performed using SPSS Statistics 22.0. Analysis of difference in protein expression between the HA group and the CA group were performed using a t-test; *p < 0.01* was set as value to indicate statistical significance.

## Supplementary information

**Additional file 1: Supplementary Table 1.** The significantly changed protein in hypothalamus of pigs under HS.

**Additional file 2:** Additional figure 1.

## Data Availability

The datasets used and/or analyzed during the current study are available from the corresponding author upon reasonable request.

## Abbreviations

17β-HSD: 17β-Hydroxysteroid dehydrogenases
ANXA: Annexins
DEPs: Differentially expressed proteins
HS: Heat stress
HSP: Heat shock protein
IPA: Ingenuity pathway analysis
iTRAQ: Isobaric tags for relative and absolute quantification
LC-MS/MS: Mass spectrometry
