# Supplementary material for: Proteomic study of hypothalamus in pigs exposed to heat stress
Source: BMC Vet Res. 2020 Aug 12;16:286. doi: 10.1186/s12917-020-02505-1 (PMC7424663; doi:10.1186/s12917-020-02505-1)
Supplement: Supplementary file 1 — Additional file 1: Supplementary Table 1. The significantly changed protein in hypothalamus of pigs under HS. [file 12917_2020_2505_MOESM1_ESM.docx]

| Supplementary Table 1 The significantly changed protein in hypothalamus of pigs under HS | | | | |
| --- | --- | --- | --- | --- |
| Protein name | Accession number | Peptides | Ratio  (Heat stress/Control) | Functions |
|  |  |  |  |  |
| *Down-regulated in Hypothalamus* | | | | |
| Similar to V-type proton ATPase subunit F-like | gi\|311275455 | 4 | 0.643 | hydrogen ion transporting ATP synthase activity |
| Similar to microtubule-associated protein tau-like | gi\|350590233 | 11 | 0.152 | microtubule binding |
| Similar to neurofilament heavy polypeptide | gi\|311270880 | 9 | 0.493 | - |
| Similar to dynamin-1 isoform 1 | gi\|194033645 | 8 | 0.333 | phospholipid binding |
| Similar to cytoplasmic dynein 1 heavy chain 1 | gi\|350587231 | 12 | 0.425 | protein binding and microtubule motor activity |
| Similar to microtubule-associated protein 6 | gi\|350588280 | 11 | 0.27 | - |
| Similar to annexin A6-like | gi\|335304211 | 3 | 0.611 | calcium ion binding |
| Similar to clathrinid coat assembly protein AP180 | gi\|350578374\|ref\|XP_003121434.3\| | 5 | 0.21 | binding |
| Similar to ankyrin-2 | gi\|350587861 | 15 | 0.543 | spectrin binding |
| Similar to dihydropyridines- related protein 1 | gi\|350587318 | 9 | 0.135 | hydrolase activity, acting on carbon-nitrogen (but not peptide) bonds |
| Similar to alpha-caltractin | gi\|194041937 | 5 | 0.52 | ATP binding |
| Similar to beta-adducin isoform 1 | gi\|335285320 | 8 | 0.381 | calmodulin binding |
| Muscle glycogen phosphorylase | gi\|106073338 | 4 | 0.329 | glycogen phosphorylase activity |
| RAB10, member RAS oncogene family | gi\|340007402 | 3 | 0.455 | GTP binding |
| Similar to tubulin beta-2C chain-like | gi\|335281298 | 18 | 0.331 | GTP binding and MHC class I protein binding |
| AP-2 complex subunit beta | gi\|342187276 | 9 | 0.444 | protein transporter activity |
| Similar to prefoldin subunit 5-like isoform 1 | gi\|335287837 | 2 | 0.492 | transcription corepressor activity |
| Similar to septin-7-like isoform 1 | gi\|311275636 | 5 | 0.368 | protein binding and structural molecule activity |
| Glia maturation factor beta | gi\|346986485 | 2 | 0.401 | protein kinase inhibitor activity |
| Similar to platelet-activating factor acetyl hydrolase IB subunit beta-like | gi\|311263970 | 2 | 0.509 | 1-alkyl-2-acetylglycerophosphocholine esterase activity |
| Similar to creatine kinase U-type, mitochondrial-like isoform 1 | gi\|311244870 | 9 | 0.284 | creatine kinase activity |
| paralemmin-1 | gi\|84042928 | 7 | 0.356 | D3 dopamine receptor binding |
| Similar to dihydropyridines- related protein 3-like | gi\|335283727 | 1 | 0.442 | chondroitin sulfate binding |
| Extracellular signal-regulated kinase-2 | gi\|310789265 | 3 | 0.291 | RNA polymerase II carboxy-terminal domain kinase activity |
| Proteolipid protein | gi\|5679718 | 6 | 0.085 | structural constituent of myelin sheath |
| Acylphosphatase | gi\|353524 | 2 | 0.398 | acylphosphatase activity |
| RTN4-Aw | gi\|38488990 | 4 | 0.266 | - |
| Heat shock 70 kDa protein 12A | gi\|350593095 | 7 | 0.439 | ATP binding |
| Peptidyl-prolyl cis-trans isomerase D | gi\|346986322 | 3 | 0.559 | peptide binding and heat shock protein |
| Unnamed protein product | gi\|1900 | 5 | 0.428 | sodium: potassium-exchanging ATPase activity |
| Similar to guanine nucleotide-binding protein G(I)/G(S)/G(O) subunit gamma-2-like isoform 3 | gi\|311245496 | 2 | 0.293 | signal transducer activity |
| Glyceraldehyde-3-phosphate dehydrogenase; | gi\|2506441 | 10 | 0.664 | NAD binding and microtubule binding |
| Alpha-soluble NSF attachment protein | gi\|346986315 | 5 | 0.537 | aspartic-type endopeptidase activity |
| Similar to uncharacterized membrane protein C1orf95-like | gi\|335296122 | 1 | 0.367 | - |
| Similar to rab GDP dissociation inhibitor alpha-like isoform 1 | gi\|335306783 | 13 | 0.293 | GTPase activator activity |
| Similar to septin-6-like isoform 2 | gi\|311276869 | 4 | 0.147 | GTP binding |
| Parvalbumin | gi\|225877918 | 2 | 0.625 | calcium ion binding |
| Similar to microtubule-associated protein RP/EB family member 3-like | gi\|350582630 | 2 | 0.192 | microtubule binding and small GTPase regulator activity |
| Similar to complexin-1-like | gi\|335293102 | 2 | 0.226 | neurotransmitter transporter activity |
| Similar to neuroplastin, partial | gi\|350586844 | 1 | 0.299 | cell adhesion molecule and 1 fibroblast growth factor receptor binding |
| Lipotropin gamma | gi\|229326 | 4 | 0.322 | - |
| Metallothionein-III | gi\|2073002 | 2 | 0.195 | - |
| Similar to V-type proton ATPase subunit E 1 isoform 2 | gi\|350584473 | 6 | 0.513 | hydrogen-exporting ATPase activity, phosphorylative mechanism |
| Rab-3A | gi\|115394766 | 2 | 0.116 | protein C-terminus binding |
| Similar to annexin A6-like | gi\|350594505 | 10 | 0.611 | calcium ion binding |
| Similar to V-type proton ATPase 116 kDa subunit a isoform 1-like isoform 1 | gi\|350590297 | 8 | 0.52 | hydrogen ion transmembrane transporter activity |
| Unnamed protein product | gi\|1921 | 4 | 0.464 | lutropin-choriogonadotropic hormone receptor binding |
| Similar to gamma-enolase isoform 1 | gi\|335288522 | 15 | 0.271 | magnesium ion binding and protein heterodimerization activity |
| Similar to protein S100-B-like | gi\|335310426 | 4 | 0.174 | kinase inhibitor activity |
| Similar to secernin-1-like | gi\|311275688 | 4 | 0.415 | dipeptidase activity |
| Similar to synaptic vesicle membrane protein VAT-1 homolog | gi\|350584838 | 9 | 0.639 | oxidoreductase activity |
| Similar to microtubule-associated protein 1A | gi\|350578745 | 11 | 0.423 | - |
| Similar to endophilin-A2-like | gi\|311248396 | 1 | 0.523 | Phosphatase, lipid and GTPase binding |
| Similar to ADP/ATP translocase 1-like isoform 2 | gi\|311272309 | 8 | 0.612 | ATP:ADP antiporter activity |
| fascin | gi\|225382133 | 6 | 0.24 | - |
| Similar to glycogen phosphorylase, brain form-like, partial | gi\|335310649 | 7 | 0.665 | glycogen phosphorylase activity and pyridoxal phosphate binding |
| Thymosin beta-4 | gi\|85700161 | 1 | 0.572 | actin binding |
| Similar to tubulin alpha-1D chain | gi\|194043861 | 17 | 0.121 | GTP binding and protein heterodimerization activity |
| Prefoldin subunit 6 | gi\|297747282 | 3 | 0.516 | chaperone binding |
| Similar to tubulin alpha-4A chain | gi\|335303414 | 18 | 0.161 | protein binding and GTP binding |
| OTUB1 | gi\|167888450 | 3 | 0.577 | omega peptidase activity |
| Aspartate aminotransferase, cytoplasmic | gi\|112976 | 5 | 0.562 | phosphatidylserine decarboxylase activity |
| Protein carboxyl-o-methyltrans ferase | gi\|19070126 | 2 | 0.259 | protein-L-isoaspartate (D-aspartate) O-methyltransferase activity |
| Similar to protein-arginine deiminase type-2-like | gi\|335290579 | 9 | 0.563 | protein-arginine deiminase activity |
| Myelin P2 protein | gi\|297307127 | 4 | 0.647 | cholesterol binding and transporter activity |
| Similar to tubulin polymerization-promoting protein family member 3-like isoform 1 | gi\|311257146 | 4 | 0.349 | tubulin binding and calcium ion binding |
| Similar to transcriptional activator protein Pur-alpha isoform 1 | gi\|335283572 | 5 | 0.629 | translation repressor activity, nucleic acid binding |
| NADP-dependent malic enzyme, mitochondrial | gi\|346716344 | 6 | 0.579 | metal ion binding and NAD binding |
| Similar to bolA-like protein 2-like, | gi\|335307557 | 1 | 0.653 | Signal transduction mechanisms; |
| Cofilin 2 | gi\|67634029 | 3 | 0.614 | actin binding |
| Na+/K+ transporting alpha 3 polypeptide | gi\|283443672 | 25 | 0.282 | metal ion binding and sodium:potassium-exchanging ATPase activity |
| Similar to spectrin alpha chain, brain | gi\|311246557 | 51 | 0.402 | calcium ion binding and spectrin binding |
| Excitatory amino acid transporter 1 | gi\|346986408 | 1 | 0.32 | high-affinity glutamate transmembrane transporter activity |
| Similar to protein arginine N-methyltransferase 1 isoform 1 | gi\|335290012 | 1 | 0.621 | histone methyltransferase activity (H4-R3 specific) |
| Similar to 26S proteasome non-ATPase regulatory subunit 12-like | gi\|350590194 | 2 | 0.56 | Posttranslational modification, protein turnover, chaperones; |
| Similar to dihydropyrimidinase- related protein 5 | gi\|311252980 | 4 | 0.371 | hydrolase activity, acting on carbon-nitrogen (but not peptide) bonds, in cyclic amides |
| Similar to syntaxin-1A-like | gi\|335284236 | 3 | 0.267 | myosin head/neck binding and SNAP receptor activity |
| Similar to syntaxin-1B-like | gi\|350581587 | 5 | 0.212 | extracellular-glutamate-gated ion channel activity |
| Mitochondrial NAD+isocitrate dehydrogenase 3 beta variant 1 | gi\|98283612 | 2 | 0.504 | NAD binding and isocitrate dehydrogenase (NAD+) activity; |
| Similar to cysteine and glycine-rich protein 1-like isoform 1 | gi\|335296245 | 4 | 0.48 | protein binding and zinc ion binding |
| Beta-synuclein | gi\|144227406 | 6 | 0.228 | calcium ion binding and alpha-tubulin binding |
| Synapsin Ib | gi\|212525788 | 13 | 0.205 | ATP binding and actin binding |
| HPCA | gi\|115394790 | 7 | 0.151 | actin binding and calcium ion binding |
| Similar to mitochondrial 2-oxoglutarate/malate carrier protein | gi\|335298430 | 6 | 0.623 | transporter activity |
| Similar to transgelin-3-like | gi\|350591990 | 5 | 0.165 | Cytoskeleton ; |
| Putative V-ATPase G subunit | gi\|6624727 | 2 | 0.203 | hydrogen-exporting ATPase activity, phosphorylative mechanism |
| Similar to protein phosphatase inhibitor 2-like | gi\|335300158 | 2 | 0.417 | protein serine/threonine phosphatase inhibitor activity |
| Galectin-1 | gi\|47716872 | 7 | 0.431 | signal transducer activity and laminin binding |
| Heat shock 90kD protein 1, beta | gi\|346986428 | 15 | 0.666 | GTP, CTP binding and UTP binding, chaperones |
| Similar to 14-3-3 protein eta | gi\|194043292 | 5 | 0.252 | sodium channel regulator activity |
| Similar to protein kinase C and casein kinase substrate in neurons protein 1-like | gi\|335292069 | 7 | 0.179 | cytoskeletal protein binding |
| Protein,myelin basic | gi\|224358 | 4 | 0.083 | structural constituent of myelin sheath |
| Similar to alpha-internexin | gi\|350593043 | 11 | 0.28 | structural constituent of cytoskeleton |
| Protein phosphatase 1 regulatory subunit 1B | gi\|2499741 | 4 | 0.225 | receptor binding and protein kinase inhibitor activity |
| Similar to synaptic vesicle glycoprotein 2A | gi\|194036298 | 6 | 0.325 | Receptor and transmembrane transporter activity |
| hexokinase 1 | gi\|342187282 | 15 | 0.637 | hexokinase activity |
| CDC37 cell division cycle 37 protein | gi\|51870491 | 5 | 0.608 | Hsp90 protein binding - |
| Similar to contactin-1-like isoform 2 | gi\|350584500 | 12 | 0.343 | glycoprotein binding and carbohydrate binding |
| HN1 | gi\|54151071 | 1 | 0.387 | - |
| Prostaglandin D synthase | gi\|1064940 | 1 | 0.443 | retinoid binding and prostaglandin-D synthase activity |
| Endorphin gamma | gi\|229600 | 2 | 0.202 | - |
| RTN4-C | gi\|38327590 | 1 | 0.31 | protein complex binding |
| Similar to cell adhesion molecule 3 | gi\|350583264 | 2 | 0.212 | protein homodimerization activity |
| somatostatin | gi\|46850198 | 2 | 0.44 | hormone activity |
| Rho GDP dissociation inhibitor alpha | gi\|315321426 | 4 | 0.625 | Rho GDP-dissociation inhibitor activity |
| Similar to LanC lantibiotic synthetase component C-like 1 | gi\|335307003 | 2 | 0.444 | G-protein coupled receptor activity |
| Similar to synaptotagmin-1 | gi\|350584732 | 4 | 0.235 | 1-phosphatidylinositol and metal ion binding |
| Similar to myosin-10-like | gi\|350590878 | 26 | 0.503 | nucleotide binding |
| Similar to 14-3-3 protein beta/alpha isoform 1 | gi\|194044626 | 6 | 0.352 | transcription corepressor activity and histone deacetylase binding |
| Similar to dynactin subunit 2-like isoform 3 | gi\|335310032 | 8 | 0.579 | motor activity |
| Similar to gamma-soluble NSF attachment protein-like | gi\|350596353 | 3 | 0.416 | - |
| peptidyl-Pro cis trans isomerase | gi\|226256 | 6 | 0.629 | Posttranslational modification, protein turnover, chaperones ; |
| Similar to endophilin-A1-like | gi\|311245746 | 2 | 0.209 | identical protein binding and lipid binding |
| Dihydrolipoamide acetyltransferase | gi\|14587786 | 4 | 0.62 | dihydrolipoyllysine-residue acetyltransferase activity |
| Similar to ubiquitin-conjugating enzyme E2 N-like | gi\|335288894 | 4 | 0.649 | ubiquitin binding and ubiquitin-protein ligase activity |
| Similar to neurofilament medium polypeptide-like isoform 1 | gi\|350592300 | 24 | 0.497 | structural molecule activity |
| Tubulin polymerization promoting protein p25 alpha | gi\|170178280 | 7 | 0.225 | microtubule binding and calcium ion binding |
| Similar to rap1 GTPase-GDP dissociation stimulator 1 | gi\|350587954 | 4 | 0.281 | GTPase activator activity |
| Tubulin beta-2B chain | gi\|343478189 | 16 | 0.154 | structural molecule activity |
| Clathrin heavy chain | gi\|224492556 | 38 | 0.362 | ankyrin binding and structural molecule activity |
| Gamma-synuclein | gi\|132269870 | 7 | 0.548 | - |
| FKBP1A-like | gi\|61098747 | 3 | 0.628 | type I transforming growth factor beta receptor binding |
| Similar to astrocytic phosphoprotein PEA-15 | gi\|194035847 | 5 | 0.262 | protein kinase C binding |
| Stathmin-1 | gi\|49615355 | 5 | 0.523 | signal transducer activity |
| Glutaminase | gi\|1583522 | 3 | 0.438 | glutaminase activity |
| Adaptor protein phosphotyrosine interaction PH domain and leucine zipper containing 1 | gi\|197130945 | 2 | 0.54 | phospholipid binding and protein kinease B binding |
| Similar to myristoylated alanine-rich C-kinase substrate | gi\|335279372 | 3 | 0.362 | protein kinase C binding and actin filament binding |
| Similar to erythrocyte membrane protein band 4.1-like 1 isoform 1 | gi\|335304751 | 9 | 0.601 | actin binding and structural molecule activity |
| Similar to dynamin 1 | gi\|350584292 | 3 | 0.5 | GTP binding and ubiquitin protein ligase binding |
| Similar to neural cell adhesion molecule 2 | gi\|350592100 | 2 | 0.307 | - |
| Similar to pyruvate kinase isozymes M1/M2 isoform 1 | gi\|194038728 | 17 | 0.4 | pyruvate kinase activity |
| CD90 protein | gi\|224697007 | 3 | 0.376 | GPI anchor binding and integrin binding |
| Neuromodulin | gi\|346716243\|ref\|NP_001231264.1\| | 4 | 0.1 | protein binding |
| Nucleoside diphosphate kinase A | gi\|325652098 | 4 | 0.394 | nucleoside diphosphate kinase activity |
| Similar to dihydropyrimidinase- related protein 3 | gi\|350581250 | 5 | 0.234 | chondroitin sulfate binding and phosphoprotein binding |
| Similar to NADH dehydrogenase [ubiquinone] 1 alpha subcomplex subunit 5-like | gi\|311275515 | 2 | 0.598 | oxidoreductase activity, acting on NADH or NADPH |
| Similar to MARCKS-related protein-like isoform 2 | gi\|311258852 | 2 | 0.382 | - |
| Similar to sodium/potassium-transporting ATPase subunit beta-2-like | gi\|311268313 | 2 | 0.172 | sodium: potassium-exchanging ATPase activity |
| Similar to neural cell adhesion molecule 1-like | gi\|311263926 | 10 | 0.256 | - |
| Similar to guanine nucleotide-binding protein G(I)/G(S)/G(T) subunit beta-2-like isoform 2 | gi\|311251041 | 5 | 0.474 | signal transducer activity |
| Similar to mu-crystallin homolog | gi\|335284508 | 4 | 0.439 | thyroid hormone binding and thiomorpholine-carboxylate dehydrogenase |
| Hexokinase II | gi\|90820093 | 2 | 0.482 | Carbohydrate transport and metabolism; |
| Similar to NADH dehydrogenase [ubiquinone] 1 beta subcomplex subunit 10-like | gi\|335310935 | 2 | 0.608 | /hexokinase activity and glucose binding |
| Similar to AP-2 complex subunit alpha-1 isoform 1 | gi\|311258000 | 5 | 0.416 | protein C-terminus binding |
| calbindin 2 | gi\|300827489 | 5 | 0.231 | calcium ion binding |
| ***Up-regulated protein in hypothalamus*** | | | | |
| Annexin A2 | gi\|52631987 | 14 | 2.322 | phosphatidylinositol-4,5-bisphosphate binding |
| Small acidic protein | gi\|349585075 | 1 | 2.098 | - |
| Apolipoprotein B-100 | gi\|350597081 | 3 | 2.815 | low-density lipoprotein particle receptor binding |
| Antioxidant protein 1 homolog | gi\|262036932 | 3 | 1.979 | copper-dependent protein binding and copper chaperone activity |
| Hsp27 | gi\|55668280 | 6 | 2.771 | protein kinase C binding and protein kinase C inhibitor activity |
| Nuclease-sensitive element-binding protein 1-like | gi\|350586335 | 4 | 1.745 | p53 binding and single-stranded DNA binding |
| 78 kDa glucose-regulated protein | gi\|350579657 | 22 | 1.634 | chaperone binding and unfolded protein binding |
| L-3-hydroxyacyl-CoA dehydrogenase | gi\|4454537 | 6 | 4.784 | 3-hydroxyacyl-CoA dehydrogenase activity |
| Isocitrate dehydrogenase [NADP] cytoplasmic isoform 1 | gi\|350593816 | 4 | 3.448 | NAD and receptor binding; isocitrate dehydrogenase (NADP+) |
| Histone H2B type 1-like | gi\|194039814 | 2 | 1.574 | DNA binding |
| Flavin reductase-like | gi\|335289705 | 5 | 3.028 | riboflavin reductase (NADPH) activity |
| immunoglobulin lambda-chain | gi\|164511 | 5 | 4.454 | - |
| Stress-70 protein, mitochondrial | gi\|311250237 | 16 | 2.105 | ATP binding and unfolded protein binding, testosterone 17-beta-dehydrogenase (NADP+) |
| 17-beta-hydroxysteroid Dehydrogenase 14-like | gi\|311257850 | 3 | 3.992 | estradiol 17-beta-dehydrogenase activity |
| Synaptosomal-associated protein | gi\|347800627 | 2 | 1.958 | syntaxin binding |
| Ppk 98; a protein kinase | gi\|431944 | 20 | 1.783 | virion and calcium ion binding |
| Aldehyde reductase | gi\|1185557 | 3 | 5.13 | electron carrier activity |
| Argininosuccinate synthase | gi\|335281104 | 9 | 19.913 | protein and toxin binding; argininosuccinate synthase activity; |
| Immunoglobulin heavy chain variable region | gi\|119663061 | 1 | 1.985 | - |
| Ribosomal protein L6 | gi\|56384243 | 4 | 1.956 | structural constituent of ribosome |
| Soluble epoxide hydrolase | gi\|45551399 | 2 | 3.051 | 10-hydroxy-9-(phosphonooxy)octadecanoate phosphatase activity |
| Alpha-1B-glycoprotein-like | gi\|311259609 | 4 | 3.927 | - |
| V-type proton ATPase subunit G 1 | gi\|298104120 | 2 | 3.062 | hydrogen-exporting ATPase activity, phosphorylative mechanism |
| Electron transfer flavoprotein beta subunit precursor | gi\|35384838 | 6 | 2.808 | electron carrier activity |
| Cathepsin D protein | gi\|56417363 | 8 | 4.76 | aspartic-type endopeptidase activity |
| Similar to protein disulfide-isomerase A4-like | gi\|311264773 | 11 | 1.889 | electron carrier activity and protein binding |
| Serpin H1 precursor | gi\|346421378 | 8 | 3.195 | serine-type endopeptidase inhibitor activity |
| IgG heavy chain precursor | gi\|5052050 | 8 | 5.141 | - |
| Prolyl 4-hydroxylase beta polypeptide | gi\|358009193 | 9 | 1.926 | protein disulfide isomerase activity |
| Solute carrier family 25 member 3 | gi\|255964672 | 3 | 1.595 | symporter activity |
| Similar to ezrin | gi\|350578005 | 9 | 4.869 | cell adhesion molecule binding and actin filament binding |
| Similar to calcium-regulated heat stable protein 1-like isoform 2 | gi\|350581733 | 1 | 3.533 | mRNA 3'-UTR and DNA binding |
| Similar to ribonuclease UK114-like isoform 1 | gi\|194037005 | 4 | 5.011 | endonuclease activity |
| Similar to prohibitin | gi\|3a50590415 | 6 | 1.519 | sequence-specific DNA binding RNA polymerase II transcription factor activity |
| Alternative Pig Liver Esterase | gi\|164414678 | 4 | 5.891 | hydrolase activity |
| Similar to proenkephalin-A | gi\|311253816 | 4 | 1.63 | neuropeptide hormone activity |
| Sorbitol dehydrogenase | gi\|346421435 | 3 | 2.822 | NAD binding and zinc ion binding |
| Similar to branched-chain -amino-acid aminotransferase, mitochondrial isoform 2 | gi\|335289923 | 3 | 7.182 | L-valine and L-isoleucine transaminase activity |
| Hemoglobin alpha | gi\|229176 | 3 | 3.057 | oxygen transporter activity and iron ion binding |
| Lumican precursor | gi\|343183420 | 7 | 2.116 | extracellular matrix structural constituent |
| Thioredoxin | gi\|14326453 | 3 | 1.604 | protein disulfide oxidoreductase activity |
| Transaldolase | gi\|349732238 | 5 | 1.532 | sedoheptulose-7-phosphate:D-glyceraldehyde-3-phosphate |
| Lysosome membrane protein 2 precursor | gi\|346421419 | 4 | 2.332 | receptor activity and enzyme binding |
| Bcl-2-like protein 13 | gi\|350584479 | 4 | 2.387 | cysteine-type endopeptidase activator activity involved in apoptotic process |
| Glycerol-3-phosphate dehydrogenase 1 | gi\|283765396 | 3 | 2.003 | NAD binding and glycerol-3-phosphate dehydrogenase activity |
| Similar to transgelin-2-like isoform 1 | gi\|335286672 | 6 | 5.511 | protein binding |
| protein SET | gi\|346644699 | 3 | 2.047 | Histone and DNA binding, protein phosphatase inhibitor activity |
| Similar to annexin A5 | gi\|335293906 | 12 | 2.117 | calcium ion binding; binding, bridging |
| tumor protein D52 | gi\|346986324 | 4 | 1.576 | protein heterodimerization activity |
| Dihydropyrimidinase | gi\|311253507 | 3 | 3.762 | dihydropyridines activity and zinc ion binding |
| Isovaleryl Coenzyme A dehydrogenase | gi\|262204892 | 5 | 2.146 | flavin adenine dinucleotide binding |
| Sterol carrier protein 2 | gi\|262263195 | 4 | 1.851 | propanoyl-CoA C-acyltransferase activity |
| Formiminotransferase-cyclodeaminase | gi\|433003 | 2 | 1.723 | folic acid and intermediate filament binding |
| Enoyl-CoA hydratase, mitochondrial | gi\|298104076 | 8 | 2.514 | protein binding and enoyl-CoA hydratase activity |
| Long-chain acyl-CoA dehydrogenase | gi\|1695729 | 2 | 2.921 | fatty-acyl-CoA binding and palmitoyl-CoA oxidase activity |
| Similar to glyoxalase domain-containing protein 4-like | gi\|335298275 | 7 | 1.616 | Amino acid transport and metabolism ; |
| APEX nuclease 1 | gi\|210062866 | 3 | 2.152 | transcription corepressor activity |
| CArG-binding factor A | gi\|160858224 | 5 | 1.718 | sequence-specific DNA binding transcription factor activity |
| Similar to 40S ribosomal protein S24 isoform 4 | gi\|194042179 | 2 | 1.72 | Translation, ribosomal structure and biogenesis ; |
| Similar to kynurenine--oxoglutarate transaminase 3 isoform 2 | gi\|311254836 | 2 | 2.85 | kynurenine-glyoxylate transaminase activity, cysteine-S-conjugate beta-lyase activity, kynurenine-oxoglutarate transaminase activity, protein homodimerization activity |
| prohibitin 2 | gi\|343780941 | 8 | 1.594 | estrogen receptor binding |
| Similar to 3-hydroxybutyrate dehydrogenase type 2-like isoform 1 | gi\|335294000 | 3 | 3.361 | 2,3-dihydro-2,3-dihydroxybenzoate dehydrogenase activity |
| Annexin A4 | gi\|4033507 | 4 | 3.162 | phospholipase inhibitor activity |
| Similar to vimentin | gi\|335296459 | 22 | 1.906 | protein C-terminus binding, structural constituent of eye lens |
| Cytochrome b5 fragment | gi\|229384 | 2 | 6.781 | cytochrome-c oxidase activity |
| Succinyl-CoA synthetase beta-subunit, partial | gi\|164669 | 6 | 4.231 | succinate-CoA ligase (GDP-forming) activity |
| Histidine-rich glycoprotein precursor | gi\|347582595 | 5 | 1.891 | heparin binding |
| Eukaryotic translation elongation factor 1 alpha | gi\|110287842 | 9 | 1.53 | protein kinase binding and translation elongation factor activity |
| Similar to alpha-actinin-4-like isoform 2 | gi\|335289608\|ref\|XP_003355931.1\| | 13 | 2.541 | integrin binding |
| unconventional myosin | gi\|516155 | 8 | 2.048 | minus-end directed microfilament motor activity |
| aminoacylase I | gi\|1845\|emb\|CAA48565.1\| | 6 | 9.061 | metal ion binding and aminoacylase activity |
| Similar to putative aminopeptidase C13A | gi\|350587379 | 3 | 2.756 | manganese ion binding and metalloexopeptidase activity |
| Glutathione S-transferase | gi\|1185280 | 2 | 18.659 | glutathione transferase activity |
| Histidine triad nucleotide-binding protein 2, mitochondrial isoform 1 precursor | gi\|346716222 | 4 | 2.219 | hydrolase activity |
| Apolipoprotein A-I | gi\|164359 | 14 | 4.284 | phospholipid binding, phosphatidylcholine-sterol O-acyltransferase activator activity |
| Epididymal secretory protein E4 | gi\|22535477 | 2 | 1.859 | serine-type endopeptidase inhibitor activity |
| Antithrombin protein | gi\|106647532 | 5 | 3.182 | Posttranslational modification, protein turnover, chaperones ; |
| Similar to annexin A11 | gi\|194042189 | 6 | 2.757 | serine-type endopeptidase inhibitor activity |
| Aldose 1-epimerase | gi\|11611545 | 3 | 5.151 | carbohydrate binding and aldose 1-epimerase activity |
| Similar to aldehyde dehydrogenase | gi\|350597032 | 1 | 5.491 | aldehyde dehydrogenase [NAD(P)+] activity |
| family 1 member A3 |  |  |  |  |
| Similar to alpha-2-macroglobulin | gi\|311256211 | 7 | 1.728 | endopeptidase inhibitor activity |
| Apolipoprotein A-II precursor | gi\|297747304 | 2 | 5.64 | protein homodimerization activity |
| 2,4-dienoyl-CoA reductase 1 | gi\|295442674 | 3 | 3.733 | oxidoreductase activity, acting on NADH or NADPH |
| Similar to selenium-binding protein 1 | gi\|194036227 | 20 | 2.677 | selenium and protein binding |
| Monoamine oxidase A | gi\|45551418 | 4 | 1.979 | primary amine oxidase activity |
| Similar to ganglioside GM2 activator-like isoform 1 | gi\|311274101 | 1 | 14.478 | phospholipase activator activity |
| Similar to hypothetical protein LOC100038023 | gi\|311245732 | 2 | 1.724 | protein kinase bindingand structural constituent of ribosome |
| Similar to methylmalonate-semialdehyde dehydrogenase [acylating], mitochondrial | gi\|194038542 | 15 | 5.125 | thiolester hydrolase activity |
| SMP-30 | gi\|115371745 | 2 | 5.202 | zinc ion binding, enzyme regulator |
| Complement component 4A | gi\|147780441 | 2 | 1.581 | endopeptidase inhibitor activity |
| Similar to hepatoma-derived growth factor | gi\|335286747 | 7 | 2.187 | growth factor activity |
| Gastrin-binding protein | gi\|433066 | 9 | 2.614 | acetyl-CoA C-acetyltransferase activity |
| Protein canopy homolog 2 precursor | gi\|297307133 | 6 | 1.89 | protein binding |
| Alpha-1 acid glycoprotein, partial | gi\|164302 | 8 | 6.08 | - |
| Similar to glutaredoxin-related protein 5, mitochondrial | gi\|194038359 | 2 | 2.363 | metal ion binding and electron carrier activity |
| G-beta like protein | gi\|495144 | 3 | 2.11 | protein kinase C binding |
| Apolipoprotein C-III | gi\|164361 | 4 | 4.813 | phospholipid binding and lipase inhibitor activity |
| Catechol-O-methyltransferase | gi\|285818436 | 6 | 2.321 | catechol O-methyltransferase activity |
| Phosphotriesterase-related protein | gi\|3466448441177.1\| | 4 | 4.447 | hydrolase activity, acting on ester bonds |
| Chromogranin B | gi\|10121853 | 25 | 3.564 | - |
| Adipocyte fatty acid-binding protein | gi\|4160392 | 5 | 324.583 | transporter activity |
| Similar to TP53-regulated inhibitor of apoptosis 1 | gi\|350592546 | 2 | 3.42 | cysteine-type endopeptidase inhibitor activity involved in apoptotic process |
| Similar to peroxiredoxin-1 isoform 5 | gi\|311259408 | 7 | 1.631 | thioredoxin peroxidase activity |
| Similar to alpha-aminoadipic semialdehyde dehydrogenase-like | gi\|350581057 | 3 | 1.551 | betaine-aldehyde dehydrogenase activity |
| 3-hydroxyanthranilate 3,4-dioxy genase | gi\|349732262 | 2 | 3.249 | 3-hydroxyanthranilate 3,4-dioxygenase activity |
| Transgelin | gi\|346421409 | 9 | 2.033 | actin binding |
| Delta-1-pyrroline-5-carboxylate dehydrogenase, mitochondrial | gi\|356582295 | 7 | 3.568 | electron carrier activity |
| Similar to carbonic anhydrase 2 | gi\|194037097 | 6 | 1.943 | carbonate dehydratase activity |
| Leukotriene A4 hydrolase | gi\|262204898 | 3 | 3.469 | aminopeptidase activity zinc ion binding |
| Similar to histone H2A type 1 | gi\|194039812 | 2 | 1.797 | DNA binding and enzyme binding |
| Myosin light chain isoform LC17b | gi\|253578 | 7 | 2.162 | structural constituent of muscle |
| uncoupling protein 3 | gi\|4165892 | 1 | 1481.875 | oxidative phosphorylation uncoupler activity |
| Heat shock 10kD protein | gi\|30525868 | 7 | 10.249 | chaperone binding |
| Mitochondrial aldehyde dehydrogenase 2 | gi\|81295909 | 6 | 7.509 | aldehyde dehydrogenase [NAD(P)+] activity |
| Similar to cytosol aminopeptidase-like | gi\|350587377 | 2 | 2.194 | metalloexopeptidase activity |
| Similar to adipocyte plasma membrane-associated protein | gi\|335308355 | 2 | 2.602 | Arylesterase and strictosidine synthase activity |
| Similar to hemoglobin subunit beta-like | gi\|311263245 | 5 | 2.37 | oxygen transporter activity |
| Ribophorin I | gi\|9857227 | 4 | 1.592 | dolichyl-diphosphooligosaccharide-protein glycotransferase activity |
| superoxide dismutase [Mn], mitochondrial | gi\|312283580 | 3 | 1.959 | manganese ion binding |
| Similar to hypothetical protein LOC100516841 | gi\|350595403 | 3 | 2.421 | 3-hydroxyisobutyrate dehydrogenase activity |
| Proteasome activator 28 beta subunit | gi\|30315381 | 4 | 1.865 | - |
| Lon peptidase 1, mitochondrial | gi\|342349346 | 10 | 3.016 | mitochondrial heavy strand promoter anti-sense binding |
| Ig heavy chain variable VDJ region, partial | gi\|558859 | 1 | 2.341 | - |
| Glutathione peroxidase 3 precursor | gi\|169646366 | 3 | 7.594 | glutathione peroxidase activity |
| Signal sequence receptor, alpha | gi\|297632426 | 1 | 2.434 | - |
| S-adenosylhomocysteine hydrolase | gi\|40644231 | 4 | 2.286 | NAD binding and copper ion binding |
| Similar to 4-trimethylaminobutyral dehyde dehydrogenase | gi\|194036835 | 4 | 4.833 | 1-pyrroline dehydrogenase activity |
| Hexosaminidase A alpha polypeptide | gi\|169117926 | 3 | 2.462 | beta-N-acetylhexosaminidase activity |
| Similar to ig kappa chain V-II region RPMI 6410 | gi\|350582129 | 3 | 2.706 | - |
| Similar to fumarylacetoacetate hydrolase domain-containing protein 2 | gi\|311252000 | 5 | 2.18 | metal ion binding |
| Similar to acyl-coenzyme A thioesterase 6 | gi\|194038528 | 3 | 1.836 | carboxylesterase and thiolester hydrolase activity |
| Thioredoxin-dependent peroxide reductase, mitochondrial | gi\|347300323 | 3 | 3.05 | protein C-terminus binding and peroxiredoxin activity |
| Beta 2-microglobulin | gi\|309795 | 1 | 1.862 | - |
| Rho GDP dissociation inhibitor (GDI) beta | gi\|346716314 | 2 | 2.344 | GTPase activator activity |
| Glucosidase 2 subunit beta precursor | gi\|347446687 | 6 | 1.733 | phosphatidylinositol binding |
| Similar to caldesmon | gi\|311275365 | 5 | 2.353 | Calmodulin, actin and myosin binding |
| Similar to purine nucleoside phosphorylase | gi\|194038973 | 5 | 2.615 | drug and phosphate ion binding |
| Similar to fumarylacetoacetase | gi\|335292272 | 5 | 6.573 | fumarylacetoacetase activity and metal ion binding |
| Similar to eukaryotic translation initiation factor 3 subunit A | gi\|194042126 | 2 | 1.961 | translation initiation factor activity |
| Similar to apoptosis-inducing factor 1, mitochondrial isoform 1 | gi\|311276941 | 2 | 3.164 | flavin adenine dinucleotide binding |
| Similar to ES1 protein homolog, mitochondrial-like | gi\|335300836 | 4 | 1.726 | Secondary metabolites biosynthesis, transport and catabolism |
| Non-selenium glutathione phospholipid hydroperoxide peroxidase (PHGPx) | gi\|6689393 | 7 | 2.148 | glutathione peroxidase activity |
| Cytochrome b5 type B | gi\|227430316 | 2 | 5.38 | metal ion binding |
| Similar to chromobox protein homolog 3-like isoform 1 | gi\|350595422 | 2 | 1.834 | identical protein binding |
| long-chain 3-ketoacyl-CoA thiolase | gi\|6165556 | 7 | 3.162 | NAD binding and long-chain- enoyl-CoA hydratase activity |
| Similar to serpin A3-8 | gi\|350587171 | 10 | 4.428 | serine-type endopeptidase inhibitor activity |
| Similar to hydroxyacid-oxoacid transhydrogenase, mitochondrial-like | gi\|311253769 | 2 | 5.101 | hydroxyacid-oxoacid transhydrogenase activity |
| Similar to adenylate kinase 2, mitochondrial-like isoform 1 | gi\|335309396 | 4 | 9.89 | adenylate kinase activity and ATP binding |
