# Supplementary material for: Proteomic study of hypothalamus in pigs exposed to heat stress
Source: BMC Vet Res. 2020 Aug 12;16:286. doi: 10.1186/s12917-020-02505-1 (PMC7424663; doi:10.1186/s12917-020-02505-1)
Supplement: Supplementary file 2 — Additional file 2: Additional figure 1. [file 12917_2020_2505_MOESM2_ESM.pdf]

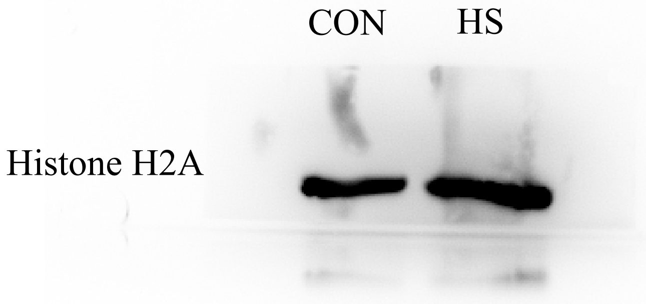


Histone H2A original image

Note： Expression of Histone H2A in the hypothalamus of pigs at day 7 of heat stress. Con = control pigs; HS = heat-stressed pigs.

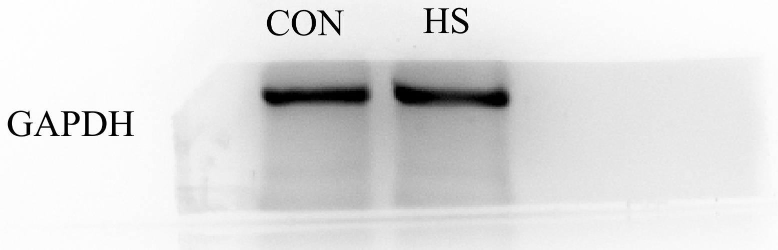


GAPDH Original image

Note： Expression of GAPDH in the hypothalamus of pigs at day 7 of heat stress. Con = control pigs; HS = heat-stressed pigs.
